# Investigation of Size-Dependent Sublimation Kinetics of 2,4,6-Trinitrotoluene (TNT) Micro-Islands Using In Situ Atomic Force Microscopy

**DOI:** 10.3390/molecules24101895

**Published:** 2019-05-17

**Authors:** Yong Joon Lee, Brandon L. Weeks

**Affiliations:** Department of Chemical Engineering, Texas Tech University, Lubbock, TX 79409, USA; yong-joon.lee@ttu.edu

**Keywords:** AFM, TNT, explosives, coarsening, sublimation, activation energy

## Abstract

Kinetic thermal analysis was conducted using in situ atomic force microscopy (AFM) at a temperature range of 15–25 °C to calculate the activation energy of the sublimation of 2,4,6-trinitrotoluene (TNT) islands. The decay of different diameter ranges (600–1600 nm) of TNT islands was imaged at various temperatures isothermally such that an activation energy could be obtained. The activation energy of the sublimation of TNT increases as the diameter of islands increases. It was found that the coarsening and the sublimation rate of TNT islands can be determined by the local environment of the TNT surface. This result demonstrates that a diffusion model cannot be simply applied to “real world” systems for explaining the sublimation behavior and for estimating the coarsening of TNT.

## 1. Introduction

Explosives have relatively low vapor pressures, making it difficult to detect traces of explosive in the air through vapor phase sampling. Other methods of detection have relied on collection of micro- or nanometer sized particles which can be subsequently heated, increasing the total explosive in the vapor phase [1,2,3]. Therefore, understanding the sublimation of explosives at given environmental conditions is crucial for the optimization of sampling and for estimating the aging/coarsening process of explosives [4]. The activation energy of sublimation of energetic materials is regarded as a very important thermodynamic parameter related to detection and aging and needs to be investigated if one is to predict the thermodynamics and aging behavior of explosives. To minimize safety risks, several studies have been recently developed using a variety of nanoscale methodologies to determine the activation energy for sublimation, including TGA, quartz crystal microbalance (QCM), UV spectroscopy, and atomic force microscopy (AFM) [5,6,7,8,9,10,11,12,13].

TGA is a well-known general method for determining the thermodynamic properties of materials. The activation energy of sublimation, and even the diffusion coefficient, of typical secondary explosives, 2,4,6-trinitrotoluene (TNT), Pentaerythritol tetranitrate (PETN), and hexahydro-1,3,5-trinitro-1,3,5-triazine (RDX), have been reported by measurement of mass loss during isothermal heating [5,6]. QCM is another tool which can be used to calculate the mass change of samples quantitatively by detecting the change in frequency of a quartz crystal resonance. It has been used to describe the sublimation process of explosives, the sublimation rate, and activation energy of sublimation for energetic materials [7,8,9]. QCM is a more accurate tool for the measurement of mass loss than TGA, but it requires high adhesion between samples and the QCM surface to achieve accurate results with high sensitivity [8]. UV absorbance spectroscopy has also been used to find the heat of sublimation of TNT, PETN, and RDX [10,11,12]. Only transparent films within a certain range of wavelengths can be used. Samples preparation is important in both QCM and UV absorbance spectroscopy analysis where thin films are normally prepared. 

AFM, on the other hand, has the advantage that it does not require a specific sample preparation. Samples can be prepared as thin films (continuous or non-continuous) or even as single crystals, depending upon the purpose of study. Moreover, it has the ability to conduct in situ imaging of surface phenomena at the nanoscale. To calculate the sublimation rate and activation energy of evaporation for PETN quantitatively, ex-situ AFM thermal analysis was first demonstrated by imaging non-continuous PETN films [13]. In other work, the activation energy of the sublimation and nucleation of PETN was obtained using in situ AFM thermal analysis with PETN single crystals [6]. While AFM thermal analysis has been performed on various explosives, surprisingly, the activation energy of TNT sublimation has not been reported. Preparation of nano-TNT is difficult because its vapor pressure is relatively higher than those of other commonly used types of secondary explosives [14].

In this report, we measured the sublimation rate and activation energy for the sublimation of various nano-sized TNT islands (600–1600 nm), using in situ AFM thermal analysis within a low temperature range (15–25 °C). Large variances in the activation energy of TNT (90–141 kJ mol^−1^) and vapor pressure have been previously reported [5,7,8,10,11,15,16,17,18,19]. This indicates that the experimentally-obtained thermodynamic parameters of TNT might vary with the condition of the system. The main goals of this study were to understand how TNT islands size affects the sublimation rate and the activation energy of sublimation. The measured sublimation rates of the TNT islands were compared to those calculated using a diffusion model [8] and values previously reported using other methods [5,10,11].

## 2. Results and Discussion

**Figure 1:**
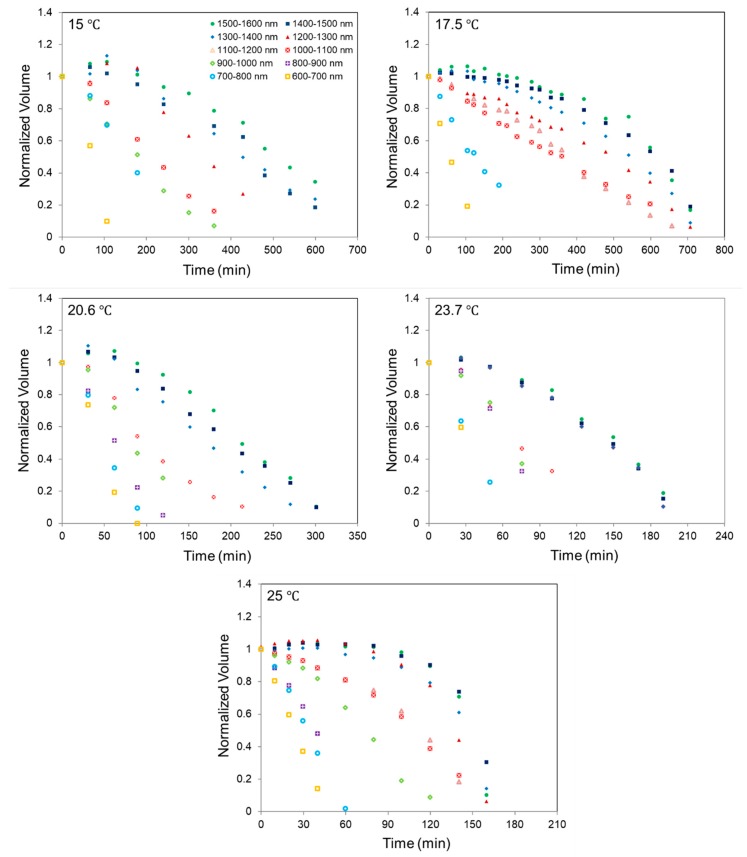
The normalized volume of various diameter range of 2,4,6-trinitrotoluene (TNT) islands as a function of time at different temperature (15, 17.5, 20.6, 23.7, 25 °C).

Figure 1 shows a series of five plots which represent the change of normalized volume for TNT islands in the various dimeter sizes (600–1600 nm) as a function of time, during annealing isothermally (15–25 °C). It was noted that the coarsening of islands at early times is typically observed at all temperatures. The decay of islands in the larger islands groups generally show non-linear shrinkage over time, whereas the volume of the smaller islands (600–800 nm) decreases nearly linearly during annealing. This might be explained by the Ostwald ripening phenomena that describes the growth of larger particles at the expense of smaller particles. Smaller particles have a higher curvature than that of a flat surface or larger particles which leads to an increase in the equilibrium vapor pressure of smaller particles [20,21]. Owing to the deviation in equilibrium vapor pressure between different size of particles, mass transfer from smaller islands to bigger islands occurs [20,21]. Therefore, the size-dependent shrinkage behavior of TNT islands on the Si-substrate can be understood as combination of two competing mechanisms, sublimation (detachment from islands) and coarsening. In the case of smaller islands, (600–800 nm) which shrink linearly, decay behavior is dominated by sublimation. Although coarsening of TNT islands is observed at low temperatures (15–25 °C), the coarsening effect decreases as temperature rises. It is reasonable to expect that the shrinkage behavior of TNT islands become dominated by the sublimation at elevated temperature ranges.

To calculate the activation energy (*E_a_*) of sublimation of TNT islands, the Arrhenius relation is used:

(1) $$-\frac{\rho dV}{Sdt}=A\exp\left(-\frac{E_{a}}{kT}\right),$$

where ρ is the density of the bulk TNT (1.654 g/cm^3^), *V* is the volume of the islands, *S* is the initial surface area of the islands before annealing, *t* is the measured time, *A* is pre-exponential factor, *k* is Boltzmann’s constant, and *T* is the absolute temperature (*K*). Each volumetric sublimation rate of various diameter size of TNT island at five different temperature (15, 17.5, 20.6, 23.7, 25 °C) is obtained by the measurement of slope of plots after the initial coarsening is terminated. Calculated volumetric sublimation rates in each size range represents average values of 1–3 different islands in the same scanned area. 

**Figure 2:**
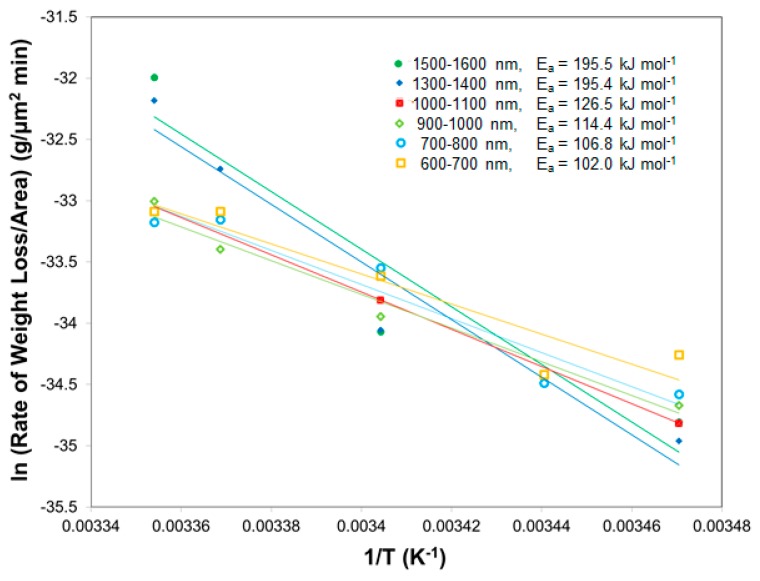
Arrhenius plot for TNT islands in the groups of various diameter size (600–1600 nm). The activation energy (*E_a_*) of sublimation of TNT islands is calculated from the slope of each Arrhenius fit.

Figure 2 shows the Arrhenius plot for different size TNT islands over the temperature range of 15–25 °C. It was observed that the activation energy of sublimation increases as the diameter of TNT islands increases. For the groups of small islands in the diameter range of 600–800 nm, the values of activation energy were calculated to be 104.4 ± 2.4 kJ mol^−1^ which corresponded to the values in the lower end of range among those reported in the previous literature (90–141 kJ mol^−1^) [5,7,8,10,11,15,16,17,18,19]. Within the middle size range (900–1100 nm), higher values of the activation energy were obtained (120.5 ± 6.1 kJ mol^−1^). In the groups of large diameter size over the 1300 nm, 195.5 ± 0.05 kJ mol^−1^ were calculated, which was considerably higher than reported value, but no trend was observed between this diameter range of islands (1300–1600 nm). One might argue that interfacial interaction between native silicon oxide (silica) on the Si-wafer and TNT islands might contribute to the discrepancy of activation energy depending upon the size of islands [7]. However, this argument might be not reasonable since the island height is greater than a few hundred nanometers. 

**Figure 3:**
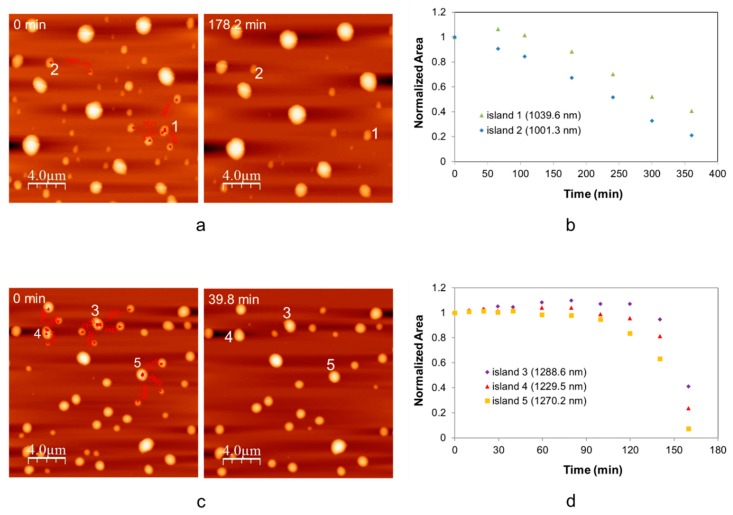
(**a**) Atomic force microscopy (AFM) height images (20 µm × 20 µm) of TNT islands at 0 min and 178.2 min during annealing at 15 °C, (**b**) the plot of normalized area versus time for island 1 and 2 at 15 °C, (**c**) AFM height images of TNT islands (20 µm × 20 µm) at 0 min and 39.8 min during annealing at 25 °C, and (**d**) the plot of normalized area versus time for island 3, 4, and 5 at 25 °C.

To investigate how the distance between islands affects the sublimation rate and the activation energy, decay behavior of two similar diameter size of TNT islands (~1 µm) with different neighbor distances during annealing at 15 °C were compared. In Figure 3a, island 1 is surrounded by smaller particles which might act as sources of coarsening, whereas island 2 is relatively isolated with larger neighboring particles. The plots of normalized area versus time for two similar diameter sizes of particles are shown in Figure 3b. The coarsening was observed only in island 1 at early periods of time. Afterward, the discrepancy of shrinkage rate of area between two particles slightly increases until the time (178.2 min) when the smaller neighbor islands nearly disappear, and then the sublimation rate seems to remain constant. The same observation is also found at 25 °C, as seen in Figure 3c,d. At the early time, coarsening is commonly observed in all three similar diameter size islands (1200–1300 nm). However, the difference of area shrinkage rate between three islands associated with local environment gradually increases with time. After 39.8 min, the coarsening of island 3 and 4 was still observed, while the area shrinkage rate of island 5 starts increasing because some neighbor smaller islands have been already consumed. Subsequently, shrinkage of the three islands becomes nearly equivalent after coarsening is terminated. 

**Figure 4:**
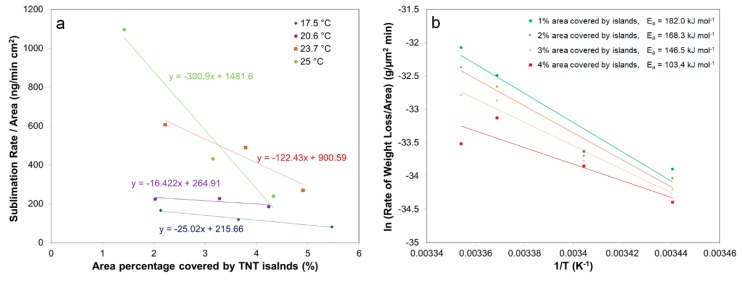
(**a**) Volumetric sublimation rate vs. area percentage covered by TNT islands (17.5, 20.6, 23.7, 25 °C) and (**b**) Arrhenius plot for TNT islands depending on total area percentage covered by TNT area in scanned area.

Figure 3d shows that the area shrinking of three islands increases, as a function of time, after coarsening is eliminated. We postulated that an increase in the sublimation rate is related to a decrease in local density of TNT islands. To validate our hypothesis, the relation between volumetric sublimation rate of total TNT islands in the scanned area, and average percentage covered by TNT islands, was analyzed at different temperatures (17.5, 20.6, 23.7, 25 °C) (Figure 4a). This was based on the assumption that local vapor density of TNT islands is associated with the surface area coverage of TNT islands on the silicon substrate. Figure 4a shows that the reduction rate in area covered by TNT islands with time, increases as temperature rises, and the sublimation rate is directly affected by the local density at higher temperature. Consequently, the activation energy of sublimation of TNT islands increases as surface area of TNT islands decreases, as observed in Figure 4b. This corresponds well with the deviation observed in activation energy of sublimation depending on diameter size of TNT islands. The increase in activation energy of sublimation with increasing size of TNT islands can be understood as a result of decrease in local density of TNT islands with time. Since small islands with diameter range of 600–800 nm are evaporated and disappeared quickly, their sublimation behavior seems to be independent on the changes of island local density during heating. 

The sublimation process of hemispherical shaped islands in an open environment can be described with a previously described diffusion model [8,9]. This model assumes the quasi stationary steady state and ideal gas state where the mass sublimation rate per area of TNT islands is given by:

(2) $$-\frac{dm}{sdt}=\frac{2\pi MDP_{sat}r_{0}}{A_{0}kT}$$

where $\frac{dm}{sdt}$ is the rate of mass loss per area (kg m^−2^ s^−1^), *M* is the molecular mass of TNT, *D* is the diffusion coefficient, *P_sat_* is the vapor pressure, *r*_0_ is the radius of islands, *A*_0_ is the initial surface area of the islands before annealing, *k* is Boltzmann’s constant, and *T* is the absolute temperature (*K*) [8,9]. To calculate the sublimation rate, two unknown parameters, vapor pressure and the diffusion coefficient, are required. Table 1 shows the reported vapor pressure data for the temperature range of 15–25 °C, calculated from the Clausius–Clapeyron equation [15,16,18,19]. The average value of data reported by Lenchitz et al. and Pella et al. is selected. For the diffusion coefficient, theoretical value, *D* = 5.59 × 10^−6^ m^2^s^−1^, is determined from literature, and the temperature variance is ignored [5,6,22,23].

Table 2 shows the comparison of the experimentally measured mass sublimation rate per area of TNT islands, those calculated from diffusion model and extrapolated values for the temperature range of 15–25 °C from other literature. As seen in Table 2, measured values are 1–2 orders of magnitude smaller than those calculated by the diffusion model. The discrepancy of two values decreases as diameter size of islands increases, and the values tend to converge at higher temperature. In addition, measured mass sublimation rate per area of TNT islands are 2–3 orders of magnitude larger than those reported by Walid et al. using other techniques, UV spectroscopy and TGA [5,10]. It is noted that in their experiment, continuous nanofilms and powders were used as samples, which are expected to have a higher local vapor density of TNT. This corresponds to our argument, as discussed earlier, that sublimation rate of TNT increases as local vapor density decreases.

Since there are limitations on collecting valid data for the sublimation rate normalized to surface area for TNT, a comparison was made to PETN, where more data is available, in Table 3, to validate our argument [6,12,13]. To be compared, all values are extrapolated for the temperature range of 30 and 40 °C. It is shown that the magnitude of mass loss rate per area are divided in to two group. In the lower sublimation rate group, non-continuous islands are used as a samples, whereas continuous film and single crystals are used as a sample in the higher mass loss rate group. Similar to TNT, the same tendency shows a correlation between sublimation rate per unit area and the local vapor density is found.

In the previous work by Burnham et al., there are two orders of magnitude difference in the evaporation rate between those measured by TGA and AFM [6]. The authors explain the discrepancy occurs due to surface migration of PETN molecules or surface quality but state that the absolute mechanism is unclear [6]. Our result could be evidence that illustrates the significant difference in sublimation rate, and the activation energy for explosives is dependent on the experimental method and types of sample used.

## 3. Experimental Section

**Figure 5:**
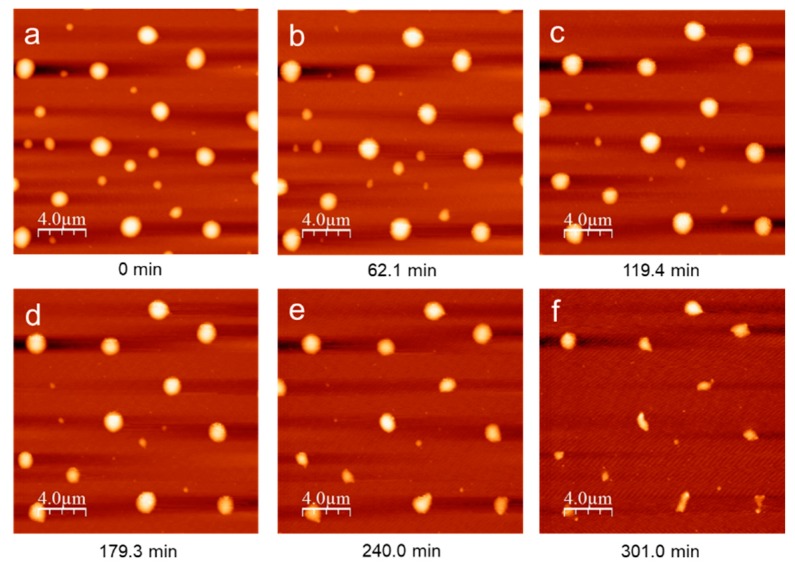
Time series of AFM height images for TNT islands during annealing at 20.6 °C. All images are 20 µm × 20 µm.

TNT powder, provided by Austin explosive, was dissolved in acetone at room temperature to prepare 0.01 M TNT solution. The solution was filtered with 0.45 µm syringe filter, and the purity of TNT was measured to be 99.67% using HPLC (BDS hypersil C-18, column size: 150 × 2.1 mm). 1 cm × 1 cm silicon wafer was cleaned by the general Piranha solution method (cleaning agent with a ratio of 3:1 sulfuric acid to hydrogen peroxide) and then, 20 µL of 0.01 M TNT solution was deposited on the Si wafer at 4000 rpm for 1 min using spin-coater (single wafer spin processor, Laurell Technologies corp., North Wales, PA, USA). At these conditions, the morphology of the TNT film typically show non-continuous hemispherical islands, and the size of particles varied with the diameter range of 0.2 µm to 1.8 µm (Figure 5). The prepared film was directly moved to the temperature stage on the AFM (XE 100, PSIA, Santa Clara, CA, USA). A conventional Peltier controller was used for in situ isothermal AFM analysis at ambient pressure open to the environment. AFM images during isothermal heating at five different temperature (15, 17.5, 20.6, 23.7, 25 °C) were collected as a function of time. In order to prevent the tip impacting the morphology of TNT islands, each image was scanned in non-contact mode with a silicon cantilever with a drive frequency of ~240 kHz imaging a 20 µm × 20 µm area. Collected images were analyzed with the Scanning probe image processor (SPIP) v. 6.7.0 (Image Metrology) and WSXM 5.0 v. 8.4 software [24].

## 4. Conclusions

In this study, we used in situ AFM thermal analysis to observe the decay behavior of nanoscale TNT islands. We observed, for the first time, that coarsening of nanoscale TNT islands occurs in the temperature range of 15–25 °C, and we showed that this coarsening effect can be affected by the local distribution of TNT islands. The activation energies calculated for the sublimation of small TNT islands, which are 600–1100 nm in diameter agree well with the values reported in the literature. Due to the significant change in local vapor density when TNT is annealed, however, the activation energies of large diameter TNT islands (1300–1600 nm) was found to be significantly higher than values in the literature. Our results are supported when the sublimation rates of TNT and PETN are compared using various types of samples with different local densities. 

## Figures and Tables

**Table 1:** Comparison of reported vapor pressure data of TNT at different temperature.

	Heat of Vaporization (kJ mol^−1^)	Vapor Pressure at 15 °C (Pa)	Vapor Pressure at 20.6 °C (Pa)	Vapor Pressure at 25 °C (Pa)
**Lenchitz et al. [15]**	103.4	2.89 × 10^−4^	6.57 × 10^−4^	1.23 × 10^−3^
**Pella et al. [18]**	99.2	2.99 × 10^−4^	6.57 × 10^−4^	1.19 × 10^−3^
**Leggett et al. [16]**	141.1	6.25 × 10^−5^	1.92 × 10^−4^	4.51 × 10^−4^
**Oxley et al. [19]**	137	6.37 × 10^−5^	1.89 × 10^−4^	4.32 × 10^−4^

**Table 2:** Comparison of mass sublimation rate per unit area for TNT at different temperature. TGA = thermogravimetric analysis.

	Sample Type	Experimental Method	Sublimation Rate at 15 °C (kg s^−1^ m^−2^)	Sublimation Rate at 20.6 °C (kg s^−1^ m^−2^)	Sublimation Rate at 25 °C (kg s^−1^ m^−2^)
**Measured**	Non-continuous micro islands	AFM	1.27 × 10^−8^ (1500–1600 nm) 1.26 × 10^−8^ (1000–1100 nm) 2.21 × 10^−8^ (600–700 nm)	2.67 × 10^−8^ (1500–1600 nm) 3.45 × 10^−8^ (1000–1100 nm) 4.20 × 10^−8^ (600–700 nm)	2.12 × 10^−7^ (1500–1600 nm) 7.52 × 10^−8^ (1000–1100 nm) 7.12 × 10^−8^ (600–700 nm)
**Calculated by Diffusion theory**	-	-	1.23 × 10^−6^ (1500–1600 nm) 1.92 × 10^−6^ (1000–1100 nm) 2.99 × 10^−6^ (600–700 nm)	2.79 × 10^−6^ (1500–1600 nm) 4.18 × 10^−6^ (1000–1100 nm) 6.29 × 10^−6^ (600–700 nm)	4.96 × 10^−6^ (1500–1600 nm) 7.32 × 10^−6^ (1000–1100 nm) 1.19 × 10^−5^ (600–700 nm)
**Walid et al. [10]**	Continuous nanofilm (500–600 nm)	UV spectroscopy	1.87 × 10^−10^	4.11 × 10^−10^	7.48 × 10^−10^
**Walid et al. [5]**	powder	TGA	1.48 × 10^−11^	3.18 × 10^−11^	5.68 × 10^−11^

**Table 3:** Comparison of mass sublimation rate per unit area for Pentaerythritol tetranitrate (PETN) at different temperature.

	Sample Type	Experimental Method	Sublimation Rate at 30 °C (kg s^−1^ m^−2^)	Sublimation Rate at 40 °C (kg s^−1^ m^−2^)
**Pitchimani at al. [13]**	Non-continuous micro islands	AFM	5.31 × 10^−9^	4.39 × 10^−8^
**Burnham at al. [6]**	Nano-islands on single crystal	AFM	3.00 × 10^−10^	1.83 × 10^−9^
**Walid et al. [12]**	Continuous nano-film (~100 nm)	UV spectroscopy	4.15 × 10^−13^	2.28 × 10^−12^
**Burnham at al. [6]**	Single crystal	TGA	9.29 × 10^−13^	5.21 × 10^−12^
